# Improving Genomic Prediction with Machine Learning Incorporating TPE for Hyperparameters Optimization

**DOI:** 10.3390/biology11111647

**Published:** 2022-11-11

**Authors:** Mang Liang, Bingxing An, Keanning Li, Lili Du, Tianyu Deng, Sheng Cao, Yueying Du, Lingyang Xu, Xue Gao, Lupei Zhang, Junya Li, Huijiang Gao

**Affiliations:** Institute of Animal Sciences, Chinese Academy of Agricultural Sciences, Beijing 100193, China

**Keywords:** hyperparameters optimization, tree-structured Parzen estimator, genomic prediction, machine learning

## Abstract

**Simple Summary:**

Machine learning has been a crucial implement for genomic prediction. However, the complicated process of tuning hyperparameters tremendously hindered its application in actual breeding programs, especially for people without experience tuning hyperparameters. In this study, we applied a tree-structured Parzen estimator (TPE) to tune the hyperparameters of machine learning methods. Overall, incorporating kernel ridge regression (KRR) with TPE achieved the highest prediction accuracy in simulation and real datasets.

**Abstract:**

Depending on excellent prediction ability, machine learning has been considered the most powerful implement to analyze high-throughput sequencing genome data. However, the sophisticated process of tuning hyperparameters tremendously impedes the wider application of machine learning in animal and plant breeding programs. Therefore, we integrated an automatic tuning hyperparameters algorithm, tree-structured Parzen estimator (TPE), with machine learning to simplify the process of using machine learning for genomic prediction. In this study, we applied TPE to optimize the hyperparameters of Kernel ridge regression (KRR) and support vector regression (SVR). To evaluate the performance of TPE, we compared the prediction accuracy of KRR-TPE and SVR-TPE with the genomic best linear unbiased prediction (GBLUP) and KRR-RS, KRR-Grid, SVR-RS, and SVR-Grid, which tuned the hyperparameters of KRR and SVR by using random search (RS) and grid search (Gird) in a simulation dataset and the real datasets. The results indicated that KRR-TPE achieved the most powerful prediction ability considering all populations and was the most convenient. Especially for the Chinese Simmental beef cattle and Loblolly pine populations, the prediction accuracy of KRR-TPE had an 8.73% and 6.08% average improvement compared with GBLUP, respectively. Our study will greatly promote the application of machine learning in GP and further accelerate breeding progress.

## 1. Introduction

Genomic selection (GS) has been widely used in actual animal and plant breeding processes, which predicts the genomic estimated breeding value (GEBV) with the whole genome markers information [1]. Compared with the traditional selection methods, which relied on progeny testing, GS tremendously accelerated the breeding process by predicting GEBV with genotypes [2]. The Holstein dairy cattle of America was the first breed that applied GS to select high-fitness individuals and has continued up until the present [3]. After more than ten years of development, GS has been gradually applied to practical genetic improvement of pigs, horses, chickens, rice, and wheat, and it significantly improved breeding [4,5,6,7,8,9,10]. Genomic best linear unbiased regression (GBLUP) and BayesB are the representative methods of GS. The former directly estimated GEBV by constructing the genetic relationship matrix (GRM) using genotypes, and the latter estimated the effect value of each single nucleotide polymorphism (SNP) using the Bayesian strategy to calculate the GEBVs [3,11,12]. However, GBLUP and BayesB predicted GEBV by building a linear model with naively ignored interaction effects and epistasis, and their prediction accuracy did not meet the desired accuracy in some populations [13]. The more robust and better compatibility genomic prediction (GP) model was what the breeder had been looking for, and they had attempted to improve the prediction accuracy by constructing a nonlinear GP model by using machine learning (ML) [14,15,16]. 

ML is a computer program learning from data to build a highly accurate prediction model without human intervention or assistance. Plentiful studies have proven the dominant position of ML in building prediction models for complex projects, such as disease diagnosis, driverless vehicles, weather forecasts, and quantitative investments [17,18,19,20,21]. In addition, ML has been considered to be the most effective implementation to parse high-throughput genome data [22]. ML has been applied to reconstruct the 3D structure of the protein, GP, and the genome-wide association study, among which the application of ML to GP was the major topic of the breeders [23,24,25,26]. González-Camacho et al. [27] compared the prediction accuracy of reproducing kernel Hilbert space (RKHS), support vector regression (SVR), deep learning (DL), and random forests (RF) with Bayesian lasso (BL) in sixteen wheat datasets. The results indicated that the prediction accuracy of SVR was higher than BL, DL, and RF. Okut et al. [28] used an artificial neural network (ANN) with Bayesian regularization to predict the GEBVs of the marbling score in Angus. The results showed that the ANN performed as well as BayesCπ, and the author believed that ANN was as useful as other GP methods for animal breeding. Montesinos-López et al. [29] evaluated the prediction accuracy of a multilayer perceptron and support vector machine (SVM) with seven real datasets; the predictions of the ML methods were very competitive, and SVM was the most efficient in terms of the computational time required. Although ML’s performance might not be preeminent in the portion dataset, the overwhelming majority of breeders were still fully confident that ML was the future of GP [27,29].

Unfortunately, the sophisticated process of tuning the hyperparameters of the ML and lacking a brilliant tuning strategy obstructed the further application of ML in actual animal and plant breeding. Generally, grid search is a highly recommended trick for parameter optimization, but it relies on rich hyperparameters tuning experience and consumes a lot of time because of its nonintelligence. Another commonly used hyperparameters tuning strategy was random search (RS), which automates hyperparameters optimization. But it seeks the optimized parameters through simple multiple random attempts, which is not the ideal parameter optimization strategy for GP.

There is an interest in using the tree-structured Parzen estimator (TPE) algorithm to optimize hyperparameters, which tunes the hyperparameters automatically with a Bayesian strategy and performs well in the reported studies [30,31,32,33]. A distinctive feature of this method is that it utilizes tree-structured adaptive Parzen estimators as a surrogate, whereas the standard Bayesian optimization algorithm utilizes Kriging (i.e., Gaussian process regression [34]). This surrogate naturally handles not only continuous variables but also discrete, categorical, and conditional variables that are difficult to handle using Kriging [30]. Nguyen et al. [31] tuned the hyperparameters of long short-term memory (LSTM) with TPE. The performance of LSTM-TPE outperformed LSTM-RS in all of the datasets. Shen et al. [33] applied natural gradient boosting with TPE to predict the runoff on the monthly, weekly, and daily scales at the Yichang and Pingshan stations in the upper Yangtze River. The results showed the proposed model improved in all indicators compared to the benchmark model. The root mean square error of the monthly runoff prediction was reduced by 9% on average and 7% on the daily scale. Their studies inspired us to apply TPE to optimize the hyperparameters of ML in GP.

Therefore, we integrated KRR and SVR with TPE to simplify the progress of optimizing hyperparameters and broaden the application of ML in GP. To evaluate the performance of TPE, we compared the prediction accuracy of KRR and SVR, which optimized hyperparameters by TPE, RS, and Grid, with GBLUP in the simulation and real datasets. The Pearson correlation coefficients between the predicted GEBV and phenotypes in the validation dataset were calculated. The mean values of fifty replicates (repeat five-fold cross-validation ten times) were used to quantify the prediction accuracy.

## 2. Materials and Methods

### 2.1. Materials

***Simulation dataset***: The simulation dataset was downloaded from the public dataset, including 4000 individuals [35]. It contained 3000 reference individuals in generation 1–3 and 1000 validation individuals in generation 4. This dataset’s phenotype consisted of three simulated traits (T1, T2, and T3), and the genotypes consisted of five chromosomes with 10,000 SNPs. This dataset has been used widely to evaluate the performance of genomic prediction methods [36,37].

***Chinese Simmental beef cattle dataset***: The data on the population was provided by the Institute of Animal Science of the Chinese Academy of Agricultural Sciences. In total, 1301 Chinese Simental beef cattle individuals in this study were born between 2008 and 2020 from Ulgai, Xilingol League, and Inner Mongolia in China. The phenotypes of this dataset consisted of seven quantitative traits, which can be divided into two types: (1) growth and development traits: live weight (LW, kg) and average daily gain (ADG, kg/day) and (2) slaughter traits: net meat weight (NMW, kg), the thickness of thigh meat (TT, cm), tenderloin weight (TW, kg), and eye muscle weight (EMW, kg). Animals were genotyped with the Illumina BovineHD BeadChip, which contained 770,000 SNPs. PLINK v1.09 [38] was taken for quality control to remove animals with missing genotypes for more than 10% of SNPs, filtering out the SNPs with a minor allele frequency (MAF) lower than 5% and a call rate (CR) lower than 95, a significant deviation from the Hardy–Weinberg equilibrium *p* < 10^−6^. After quality control, 1287 individuals with 671,990 SNPs have been retained. 

***Loblolly pine dataset***: The original genotypes of the 951 individuals in the loblolly pine dataset contained 7216 SNPs [39]. After QC, 4853 SNPs were retained for the subsequent analyses in this study. Eight traits were selected to reconstruct the phenotypes of this dataset, which refers to growth, development, and disease resistance, three types of traits: crown width along the planting beds (CWAL, cm), total height to the base of the live crown (HTLC, cm), branch angle average (BA, degree), crown width across the planting beds (CWAC, cm), average branch diameter (BD, cm), gall volume (GALL), the presence or absence of rust (Rust_bin), and the basal height of the live crown (BHLC, cm). The heritability of these traits was from 0.12 to 0.45.

***Pig dataset***: All of the individuals in this dataset were genotyped with the Illumina PorcineSNP60 chip, and the SNPs were filtered with the following criteria: MAF < 0.03, genotype call rate <95%, and HWE test *p*-value < 10^−4^. Referring to the previously reported studies [40], we selected four traits (T2, T3, T4, T5) to assess the performance of each method in this study because the number of records of trait T1 was limited. 

***German Holstein population***: This dataset consisted of 5024 bulls, in which the individuals were genotyped with the Illumina Bovine SNP50 Beadchip, and three phenotypic types of information were recorded. In the QC procedure, the SNPs with a call rate less than 95%, MAF less than 0.01, and HWE (*p* < 1 × 10^−4^) were removed. The genotype data in the following analyses contained 42,551 SNPs. According to Hu et al. and Zhe et al. [41,42], three traits—somatic cell score (SCS), milk fat percentage (MFP, %), and milk yield (MY, kg)—were selected to represent different genetic architectures.

The statistical description of each dataset is shown in Table 1.

### 2.2. Methods

***TPE***: TPE was a Bayesian optimization algorithm that optimized the hyperparameters automatically. Hyperparameters optimization of TPE can be represented as:

(1) $$x^{*}=\arg\;\underset{x\in \mathbb{R}}{\min}\left(-f_{M}\left(x\right)\right)$$

where $f_{M}\left(x\right)$ represents an objective score to maximize model *M*, $x^{*}$ is the set of hyper-parameters that yields the highest score, and x can take on any values in the space ℝ. In the optimized progress of Equation (1), excepted improvement (*EI*) was used as the criterion. *EI* is the expectation under some model *M* of f:x→R that $f_{M}\left(x\right)$ will exceed some threshold $y^{*}$:

(2) $$EI_{y^{*}}\left(x\right)=\int_{-\infty }^{+\infty }\max\left(y^{*}-y,\;0\right)p_{M}(y|x)dy$$

In direct contrast to the Gaussian and random research model $p_{M}(y|x)$ are TPE models p(x|y) and p(y), where p(x|y) can be written as:

(3) $$p\left(x|y\right)=\{\begin{array}{c} l\left(x\right),\;\;\mathrm{if}\;y<y^{*}\\ g\left(x\right),\;\;\mathrm{if}\;y\geq y^{*} \end{array}$$

where l(x) is the density formed by using the observations $\left\{x^{\left(i\right)}\right\}$ such that the corresponding objective score was lower than $y^{*}$ and g(x) is the density formed by using the remaining observations.

With Bayes’ rule, the *EI* equation becomes:

(4) $$EI_{y^{*}}\left(x\right)=\int_{-\infty }^{y^{*}}(y^{*}-y)\cdot p_{M}(y|x)dy=\int_{-\infty }^{y^{*}}\left(y^{*}-y\right)\cdot \frac{p_{M}(x|y)\cdot p_{M}\left(y\right)}{p_{M}\left(x\right)}dy$$

Finally, the *EI* can be represented as:

(5) $$EI_{y^{*}}\left(x\right)=\frac{\gamma \cdot y^{*}\cdot l\left(x\right)-l\left(x\right)\cdot \int_{-\infty }^{y^{*}}p_{M}\left(y\right)dy}{\gamma \left(x\right)+\left(1-\gamma \right)\cdot g\left(x\right)}\propto {\left(\gamma +\frac{g\left(x\right)}{l\left(x\right)}\left(1-\gamma \right)\right)}^{-1}$$

**Figure 1:**
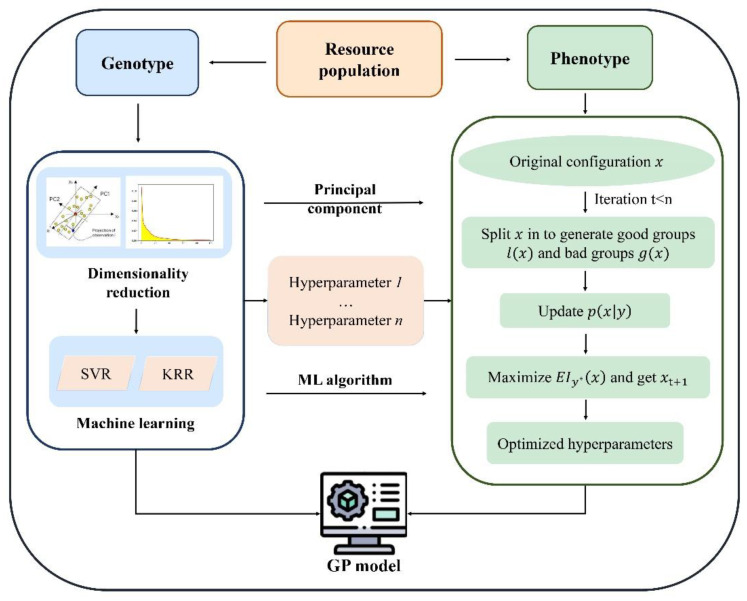
Optimization of the hyperparameters of KRR and SVR to construct the GP model using TPE. The working process can be divided into three steps: (1) select the ML algorithm and the dimension reduction methods. In this study, the ML we used was KRR or SVR, and the method to reduce the dimension was principal component analysis; (2) determine the hyperparameters that need to be optimized, which include the number of the principal component to construct the prediction model and the hyperparameters of KRR (e.g., kernel, alpha, etc.) and SVR (e.g., kernel, gamma, C, etc.); and (3) optimize the hyperparameters by using TPE and training the prediction model for GP.

Equation (5) indicated that *EI* is proportional to the ratio of l(x)/g(x), and therefore, to maximize *EI*, we should draw scores of the hyperparameters, which are more likely to be under l(x) than under g(x). The TPE works by drawing sample hyperparameters, evaluating them in terms of  l(x)/g(x), and returning the candidate $x^{*}$ greatest *EI* for each iteration. In this study, we achieved TPE with the help of a hyperopic Python package, which can be obtained at https://github.com/hyperopt/hyperopt, accessed on 29 September 2022. The process of using TPE to optimize the hyperparameters of KRR or SVR is demonstrated in Figure 1. In this study, the TPE was performed with Python software with the *hyperopt* package.

***PCA***: Principal component analysis (PCA) is a popular technique for analyzing large datasets containing a high number of dimensions per observation, increasing the interpretability of data while preserving the maximum amount of information and enabling the visualization of multidimensional data. In this study, we applied PCA to reduce the dimensionality of the input feature, and the number of (k) components incorporated in SVR and KRR was optimized by TPE. The PCA procedure was performed using the Python software with the *sklearn* package.

***KRR***: KRR utilizes the kernel trick ($\phi \left(x_{i}\right)$) to construct a newly latent feature space and then build the ridge regression model in the latent space. KRR is represented as $y_{i}=\beta^{T}\phi \left(x_{i}\right)$, where β is the vector of weights. Regularized least squares is applied to optimize β as follows:

(6) $$\min L_{\mathrm{KRR}}=\frac{1}{2}{∥\beta ∥}^{2}+\frac{1}{2}C\sum_{i=1}^{n}{\left({\hat{y}}_{i}-\beta^{T}\phi \left(x_{i}\right)\right)}^{2}$$

where *C* is the regularization constant. By taking the derivative of $L_{KRR}$ concerning *β* equating the resulting equations to zero, the output weight vector β is obtained as:

(7) $$\beta ={\left(\phi^{T}\phi +\frac{I}{C}\right)}^{-1}\phi^{T}\hat{y}\;\;$$

where $\phi \left(x_{i}\right)$ is the instance in the latent feature space. I is an identity matrix. According to the representer theorem, β can be represented with α as:

(8) $$\beta =\sum_{i=1}^{n}\alpha_{i}\phi \left(x_{i}\right)=\phi^{T}\alpha \;$$

thus:

(9) $$\alpha ={\left(\phi^{T}\phi +\frac{I}{C}\right)}^{-1}\hat{y}={\left(K+\frac{I}{C}\right)}^{-1}\hat{y}\;\;$$

where *K* is the kernel matrix whose entries are obtained as:

(10) $$K\left(x_{i},\;x_{j}\right)=\phi \left(x_{i}\right)\phi {\left(x_{j}\right)}^{T}$$

Finally, with a new test instance $x_{i}$, the predicted value is obtained using dual weights and similarity between the test sample $x_{i}$ and all the training samples used for prediction.

(11) $$y(x_{i})=k\prime {\left(K+\frac{I}{C}\right)}^{-1}\hat{y}\;\;$$

where $k^{\prime }=K\left(x_{i},\;x_{j}\right)$, *j* = 1, 2, …, n. In this study, the KRR was performed using the Python software with *sklearn* package, and the hyperparameters that need to be tuned are demonstrated in Table 2. 

***SVR****:* For regression with a continuous response, SVR is fitted by the following formula:

(12) $$f\left(x\right)=\beta_{0}+h{\left(x\right)}^{T}\beta \;$$

where $h{\left(x\right)}^{T}$ is the kernel function. As for the ‘ε-insensitive’ SVM regression, the SVR problem is represented as:

(13) $$\underset{\beta_{0},\;\;\beta }{\min}\frac{1}{2}{∥\beta ∥}^{2}+\sum_{i=1}^{n}V\left(\gamma_{i}-f\left(x_{i}\right)\right)\;\;$$

where

(14) $$V_{\varepsilon }\left(r\right)=\{\begin{array}{c} 0\;\;\;\;\\ \left|r\right|-\varepsilon \end{array}\;\;\;\;\begin{array}{c} if\;\left|r\right|<\epsilon \\ otherwise \end{array}$$

$V_{\varepsilon }\left(r\right)$ is an ‘ε-insensitive’ error measure. If the absolute error between $f\left(x_{i}\right)$ and $y_{i}$ of the *i*th instance is bigger than ε, the loss is calculated. λ is a positive constant. ${∥\cdot ∥}^{2}$ denotes the norm under a Hilbert space.

The SVR can be written as follows:

(15) $$\hat{\beta }=\sum_{i=1}^{n}\left({\hat{\alpha }}_{i}^{*}-{\hat{\alpha }}_{i}\right)x_{i},$$

and,

(16) $$f\left(x\right)=\sum_{i=1}^{n}\left({\hat{\alpha }}_{i}^{*}-{\hat{\alpha }}_{i}\right)x_{i}$$

where ${\hat{\alpha }}_{i}^{*}$, ${\hat{\alpha }}_{i}$ are positive weights given to each observation and estimated from the data, and the inner product kernel *K*($x_{i},\;x_{i}$) is an n×n symmetric and positive definite matrix. All of the SVR procedure was performed using the Python software with *sklearn* package, and the hyperparameters needed to be optimized, as shown in Table 2.

***GBLUP***: GBLUP estimates GEBVs using phenotypes and genomic relationships that are calculated with the whole genome markers’ information [43]. In GBLUP, the effect of each SNP was assumed to follow the identical normal distribution [44]. The formula of GBLUP is as follows:

(17) $$y^{*}=Z\gamma +e$$

where $y^{*}$ is the vector of the corrected phenotype, and Z is an incidence matrix for individual effects. $\gamma \sim N\left(0,\;G\sigma_{g}^{2}\right)$ is a vector of breeding values. The $\sigma_{g}^{2}$ is genetic variance. $e\sim N\left(0,I\sigma_{e}^{2}\right)$ is a vector of residuals, where I is an identity matrix and $\sigma_{e}^{2}$ is the residual variance. G matrix was calculated with the formula: $G=\frac{ZZ^{\prime }}{2\sum p_{i}\left(1-p_{i}\right)}$, $p_{i}$ is the MAF of the i-th marker. This study performed GBLUP using the software R with the *rrBLUP* package.

### 2.3. Assessing Prediction Performance

This study quantified the prediction accuracies with the Pearson correlation coefficients between predicted GEBV and the phenotypes. The prediction accuracy demonstrated in this paper was the mean of fifty replicates of ten-times repeat five-fold cross-validation for each trait. The Pearson correlation coefficient was calculated as follows: $r\left(y^{*},\;GEBV\right)=\frac{cov\left(y^{*},\;GEBV\right)}{\sqrt{var\left(y^{*}\right)var\left(GEBV\right)}}$, where $y^{*}$ was the phenotype. 

To test the statistical significance of the observed differences between the method, Friedman’s and Nemenyi post hoc tests were performed with the Python software with the *Orange* and *Scipy* package. Friedman’s test operated on the average rank of the methods and checked the validity of the hypothesis (null hypothesis) and verified that all methods were equivalent. The Nemenyi post hoc test was used to find which methods, in particular, differed from GBLUP.

## 3. Results

***Simulation dataset***: Firstly, we demonstrated the performance of KRR and SVR, tuning the hyperparameters with TPE, RS, and Grid, compared with GBLUP in the simulated dataset (Table 3). The simulated dataset, QTLMAS 16th, includes three traits, and the heritability was 0.45, 0.38, and 0.48, respectively. Table 2 summarizes the prediction accuracy performances of each strategy. The results showed that the average prediction accuracy of GBLUP is similar to KRR-TPE, and the prediction accuracy of SVR is slightly lower. For KRR, using TPE to optimize the hyperparameters achieved the highest accuracy, but KRR-RS also achieved good performance and was on average just 0.24% lower than KRR-TPE. The performance of SVR was unsatisfactory in this dataset, except for the lower prediction accuracy in T1 and T2. It can also not be applied to the GEBV prediction of T3. 

***Chinese Simmental beef cattle***: In addition to the simulation data, we also evaluated the prediction ability of each strategy in the real animal and plant dataset from cattle, cows, pigs, and pine. We compared the average prediction accuracy of each method in Table 4. The results indicated that the KRR-TPE predicted more accurate GEBV compared with KRR-RS, KRR-Grid, and GBLUP, improving prediction accuracy by 1.67% (−0.47–6.12%), 10.36% (8.73–13.08%), and 8.73% (3.40–17.88%) respectively. Although the performance of SVR did not meet the expectations compared with the reported studies, the prediction ability of SVR-TPE and SVR-RS was comparable with GBLUP for the Chinese Simmental beef cattle population.

**Figure 2:**
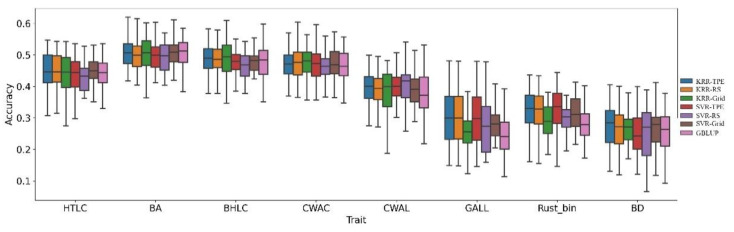
Comparison of prediction accuracy of KRR, SVR, with different hyperparameters-optimized strategies, and GBLUP for Loblolly Pine datasets. The prediction accuracy was assessed by the Pearson correlation between predicted GEBV and phenotypic values with five-fold cross-validation and was repeated ten times.

***Loblolly Pine****:* The Loblolly Pine dataset concluded eight quantitative traits and the heritability of the traits was from 0.12 to 0.43. Figure 2 demonstrates the prediction ability of each method in the Loblolly Pine dataset. The KRR-TPE achieved the highest predictive ability only in four traits (HTLC, BD, BHLC, GALL). For the other traits (BA, CWAC, CWAL, Rust_Bin), the prediction ability of KRR-TPE was almost the same as SVR-TPE, which had the highest prediction accuracy. Considering all of the traits of the Loblolly Pine dataset, KRR-TPE possessed the most powerful prediction ability, the prediction accuracy of which was 0.70% (−0.21–2.23%), 3.98% (−0.42–13.85%), 1.76% (−0.51–9.13%), 4.83% (−1.26–12.15%), 1.35% (−0.42–4.96%) and 6.08% (0.45–20.33%) higher than KRR-RS, KRR-Grid, SVR-TPE, SVR-RS, and SVR-Grid, respectively.

**Figure 3:**
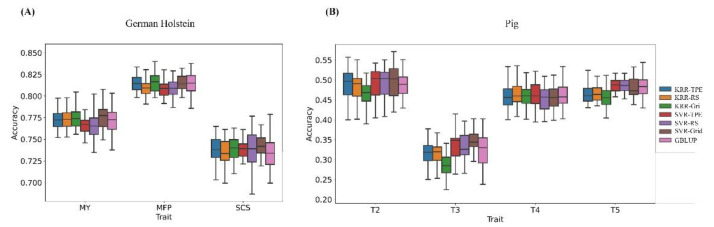
The comparison of the prediction accuracy of each method in the actual animal dataset: pig population (**A**) and German Holstein population (**B**). The prediction accuracy was assessed by the Pearson correlation coefficient between predicted GEBV and phenotypic values with five-fold cross-validation and was repeated ten times.

***German Holstein population and pig population***: Moreover, we calculated the average prediction accuracy of each method in the German Holstein population and pig population, both of which were genotyped using the chips with a marker density of about 50 k. Figure 3 compares the predictive ability of various methods to predict GEBV in the German Holstein and pig populations. For the pig population (Figure 3A), except that KRR-Grid performed worse than GBLUP, an average of 5.63%, the rest of the methods were comparable with GBLUP, and there was no method that outperformed other methods. Figure 3B reports the predictive performance of the methods in the German Holstein population, which was similar to the performance in the pig population; there was no obvious difference in the average of each method.

**Figure 4:**
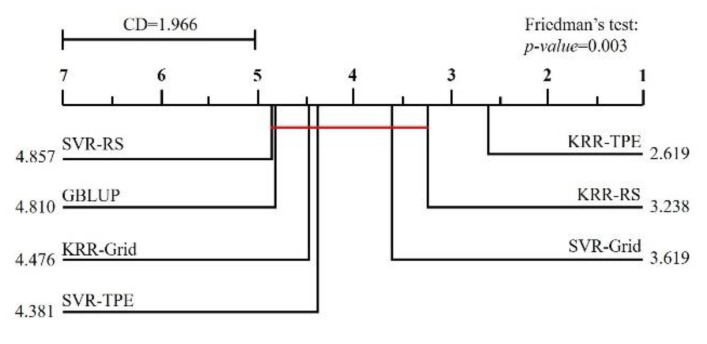
Comparison of the average rank of each method. A group of methods that do not significantly differ from GBLUP (*p* = 0.05) are connected by the red line.

**Figure 5:**
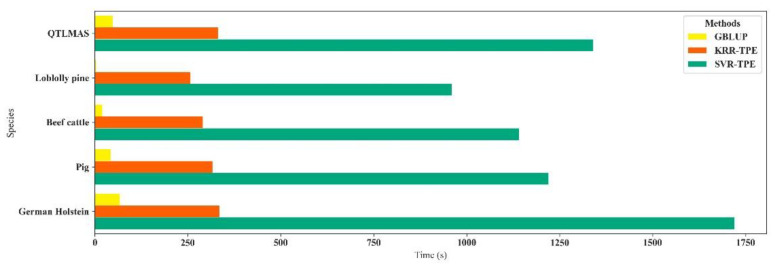
Time consumed by GBLUP, KRR-TPE, and SVR-TPE.

***General evaluation***: Firstly, we used Friedman’s test and the Nemenyi post hoc test to test the statistical significance of the observed difference in all datasets, and the testing results are shown in Figure 4. The *p*-value of Friedman’s test was 0.003, which means the average rank of each method was not equivalent. According to the Nemenyi post hoc test, the average rank of KRR-TPE differed significantly from GBLUP, and the average rank of other methods was not significantly different. Subsequently, we compared the time consumed by GBLUP, KRR-TPE, and SVR-TPE (Figure 5), and the results indicated that KRR-TPE was slower than GBLUP and faster than SVR-TPE.

## 4. Discussion

As ML has entered the twenty-first century, the theory and application of ML have been greatly developed. Breeders desired to predict more accurate GEBV with ML, further accelerating the genetic gain in animal and plant breeding. Before constructing the prediction model, the hyperparameters should be appointed, which determine whether machine learning algorithms can effectively mine information in the high-throughput genome data. The most-reported studies that used ML to predict GEBV tuned hyperparameters manually, which was cumbersome and relied on rich experience and was thus not friendly for novices. Therefore, there is an urgent need to develop an uncomplicated hyperparameters optimization strategy to promote ML application in GP. Thus, we simplified the process of predicting GEBV by using the automatic parameters tuning strategy, TPE, which optimized the hyperparameters automatically and performed better in reported studies [30,31,32,33]. The performance of KRR and SVR, tuned by TPE, RS, and Grid separately, were assessed by the simulation dataset and four real datasets. Overall, the performance of KRR-TPE outperformed the other methods. 

Typically, the grid search sets a series of initial values for each hyperparameter and then evaluates the prediction ability of the estimator with every combination to select the optimum parameters with the most robust hyperparameters configuration. Although grid search works well in most cases, it can only ensure that the parameters selected are relatively optimal because it did not search from the complete parameter space. RS is essentially similar to grid search, randomly selecting the combination from the hyperparameters space and then repeating this process. If we repeat this process enough times, we can get the best configuration of the hyperparameters. To a certain extent, RS was capable of tuning hyperparameters automatically, but it seems impractical for genomic prediction due to being time-consuming. Although grid search and RS might find the super parameters relatively suitable for the prediction model, these have an obvious disadvantage and are still not intelligent enough. 

The TPE algorithm is designed to optimize quantization hyperparameters with a more brilliant strategy to find a quantization configuration that achieves an expected accuracy target. TPE is an iterative process that uses the history of evaluated hyperparameters to create a probabilistic model using the Bayesian algorithm, which is used to advise on the next configuration of hyperparameters to evaluate. Therefore, it is highly performant and very time-efficient compared with RS and grid search [45]. Our results again proved that TPE is an excellent automatic tuning hyperparameters strategy. According to the results of the general evaluation section, the KRR-TPE was the only method significantly superior to GBLUP.

As for why the performance of SVR-TPE did not outperform SVR-Grid, we analyzed the process of tuning the hyperparameters of SVR and believe there were the following two main determinants. Firstly, to ensure the consistency of experimental results and the acceptability of calculation time for the actual breeding program, the iteration times to optimize the hyperparameters of SVR-TPE and KRR-TPE were set to only 200. However, SVR had more hyperparameters compared with KRR, and insufficient iterations might result in SVR-TPE not achieving higher prediction accuracy. Secondly, our team had previously accumulated a lot of experience in SVR hyperparameters optimization in GP. Consequently, we can easily achieve the high prediction accuracy of SVR by manually tuning hyperparameters, but it is not realistic for most breeders and experimenters. Therefore, we have reason to believe that TPE has great potential to promote the wide application of ML in GP.

Although the computing speed of computers has been significantly improved, computing speed is still a major problem to be solved when widely applying ML to GP. To further tap the potential of automatic hyperparameters tuning and promote the application of ML in actual animal breeding, running the GP program in the graphics processing unit (GPU) might be a practical tactic. From my perspective, only by combining computer science, mathematics, and biology can we fully phase high-throughput genome information and achieve a breakthrough in animal breeding.

## 5. Conclusions

In conclusion, we first integrated KRR with tree-structured Parzen estimator optimized hyperparameters automatically to simplify the process of using machine learning for genomic prediction. The results indicate that incorporating KRR and TPE can greatly and more conveniently improve the prediction accuracy of GEBV. Especially for the Chinese Simmental beef cattle and Loblolly pine populations, the prediction accuracy of KRR-TPE had an 8.73% and 6.08% average improvement compared with GBLUP, respectively. Our study also promotes machine learning’s wider application in actual animal and plant breeding.

## Figures and Tables

**Table 1:** Summary of five datasets.

Dataset	Trait	N	h^2^	Mean	SD
Simulation	T1	3000	0.36	0.00	176.52
	T2	3000	0.35	0.00	9.51
	T3	3000	0.52	0.00	0.02
Beef cattle	LW	1285	0.30	510.37	73.50
	ADG	1282	0.28	0.97	0.21
	NMW	1273	0.32	233.02	41.75
	TT	1275	0.30	17.89	2.12
	TW	1284	0.29	8.75	1.98
	EMW	1281	0.38	10.67	2.22
Loblolly pine	HTLC	861	0.31	20.30	73.31
	BA	861	0.45	2.28	42.03
	BD	910	0.11	0.05	1.20
	BHLC	861	0.35	0.092	0.51
	CWAC	861	0.45	2.28	42.03
	CWAL	861	0.27	2.44	27.33
	GALL	807	0.12	−0.022	1.31
	Rust_bin	807	0.21	−0.014	0.40
Pig	T2	2715	0.16	0.00	1.12
	T3	3141	0.22	0.71	0.96
	T4	3152	0.32	−1.07	2.33
	T5	3184	0.38	37.99	60.45
German Holstein	MY	5024	0.95	370.79	641.60
	MFP	5024	0.94	−0.06	0.28
	SCS	5024	0.88	102.32	11.73

Note: N: Number of individuals with phenotypes. h^2^: heritability. SD: standard deviation.

**Table 2:** The hyperparameters need to be tuned.

KRR		SVR	
Kernel	Cosine, RBF, Linear	Kernel	RBF, Linear, Ploy
Gamma	0.000001–0.001	Degree	1, 2, 3, 4
Alpha	0–10	Gamma	0.000001–0.001
K	1–n	C	0.1–100
		K	1–n

n: the number of the principal component of the results of PCA. Cosine: Cosine kernel, RBF: Radial basis function kernel, Linear: Linear kernel, Ploy: Polynomial kernel

**Table 3:** The average prediction accuracy of each method in the simulation dataset.

Trait	KRR			SVR						GBLUP
	TPE	RS	Grid	TPE		RS		Grid		
T1	**0.402**	0.400	0.394	0.39		0.386		0.393		0.406
T2	**0.398**	0.395	0.395	0.387		0.382		0.384		0.400
T3	**0.546**	0.541	0.544							0.545
					

Note: the prediction accuracy of each method was assessed by the Pearson correlation coefficient between the predicted GEBV and the phenotypes of each trait. Five-fold cross-validation repeated ten times was used to ensure the high credibility of the results.

**Table 4:** Comparison of prediction accuracy of each method using seven quantitative traits in the Chinese Simmental beef cattle.

Trait	KRR			SVR			GBLUP
	TPE	RS	Grid	TPE	RS	Grid	
LW	**0.298**	0.288	0.264	0.278	0.271	0.286	0.276
ADG	0.274	**0.275**	0.252	0.215	0.202	0.222	0.265
MW	**0.298**	0.287	0.27	0.268	0.271	0.262	0.270
TT	0.294	**0.295**	0.26	0.249	0.288	0.247	0.274
TW	**0.208**	0.196	0.197	0.193	0.201	0.185	0.193
EMW	0.314	**0.316**	0.288	0.291	0.286	0.27	0.295

Note: prediction accuracy performance was measured by the Pearson correlation coefficient. The prediction accuracy was calculated by five-fold cross-validation repeated ten times, which means that the prediction accuracy in Table 3 is the mean of the fifty Pearson correlation coefficients between the predicted GEBV by each method and the phenotypes.

## Data Availability

Chinese Simmental Beef Cattle dataset: Data are available from the dryad digital repository: http//DOI:10.5061/dryad.4qc06. German Holstein dataset: Data can be obtained at https://www.g3journal.org/content/5/4/615.supplemental. Pig dataset: Data are available from https://academic.oup.com/g3journal/article/2/4/429/6026060?login=true#supplementary-data. Loblolly pine dataset: The quality-controlled genotypes can be found at https://www.genetics.org/highwire/filestream/412827/field_highwire_adjunct_files/1/FileS1.zip and the complete phenotypes at https://www.genetics.org/highwire/filestream/412827/field_highwire_adjunct_files/4/FileS4.xlsx.

## Code Availability

The code in this study is available in https://github.com/ML-GS/KRR-TPE.
